# The impact of the leg-lengthening total hip arthroplasty on the coronal alignment of the spine

**DOI:** 10.1186/1748-7161-10-S2-S4

**Published:** 2015-02-11

**Authors:** Yuichiro Abe, Shigenobu Sato, Satomi Abe, Takeshi Masuda, Kentaro Yamada

**Affiliations:** 1 Department of Orthopaedic Surgery, Wajokai Eniwa Hospital, Eniwa, Hokkaido, Japan

## Abstract

**Background:**

Coronal imbalance of the pelvis is recognized to lead to the development of degenerative lumbar scoliosis. We hypothesized that an abrupt change of pelvic obliquity may show a reproducible trend of coronal compensation in the lumbosacral spine. The aim of the study was to classify the change of coronal alignment of spine after THA.

**Methods:**

This is a retrospective study based on the radiological analysis of 195 patients who underwent THA between 2009 and 2010. The mean age at surgery was 61.5 years old, and minimum follow up period was 24 months. Pelvic obliquity (POb) and Cobb’s angle of lumbar scoliosis (LS) in coronal plane were measured. Over 3.5 degrees of change in POb was regarded as ΔPOb(+) and over 10 degrees of lumbar scoliosis was regarded as LS(+). The change of LS were classified into 3 subtypes; ΔLS(+), over 5 degrees of progress in LS, ΔLS(-), over 5 degrees of improvement in LS, and ΔLS(n), changes in LS within 5 degrees.

**Results:**

Over 3.5 degrees of change in POb was significantly correlated with the change in LS. Among195 patients, 120 patients improved their pelvic obliquity (ΔPOb(+)), and 75 patients did not have an improved pelvic obliquity (ΔPOb(-)). 99 patients out of 120 ΔPOb(+) patients did not show changes (54, ΔLS(n)) or improvement in scoliosis (45, ΔLS(-)).The remaining 21 patients showed progress or development of de novo scoliosis. Patients who failed to compensate for the POb change at lumbosacral area developed de novo lumbar scoliosis (7 cases), showed progression in lumbar scoliosis (7 cases) or developed coronal trunk shift over 20mm (7 cases)

**Conclusions:**

The patterns of compensation in lumbar or lumbosacral spine in coronal plane after leg lengthening THA were classified with regards to pelvic obliquity and Cobb’s angle. 89.2% of 195 patients showed acceptable compensation in lumbar spine, 21 patients developed coronal imbalance. THA therefore is considered to be safe, as regards to spinal balance in coronal plane. However we have to keep in mind that preoperative rigid scoliosis could have a risk in progress for spinal imbalance.

## Background

Alteration of pelvic obliquity also has been reported to induce secondary lumbar scoliosis. Papaioannou [1] reported that limb-length inequality developed compensatory lumbar scoliosis in young adults, and horizontalization of tilted pelvis by leg lengthening improved these cases. In elderly patients with osteoarthritic hip joints, asymmetric pelvic also is thought to develop degenerative lumbar scoliosis. Previous reports, however, showed that there was no correlation between the degrees of pelvis elevation and magnitude of lumbar scoliosis and have failed to find out a reproducible trend [2-5]. We hypothesized that an abrupt change of pelvic obliquity may show a reproducible trend of coronal compensation in the lumbosacral spine. The aim of the study was to classify the change of coronal alignment of spine after THA.

## Methods

This is a retrospective study based on the radiological analysis of patients who underwent THA between 2009 and 2010. A total of 300 patients (270 females and 30 males) were enrolled in this study. The mean age at surgery was 61.5 years old (range 30-84), and minimum follow up period was 24 months. One hundred and five patients who had not undergone whole spine standing X-ray at pre-op or final follow up, and who received additional hip surgery or previous lumbar fusion surgery were excluded, and the remaining 195 patients were evaluated. The study was approved by the Ethical Committee for Clinical Research at Wajokai Eniwa Hospital (approval date: April 10 2011).

### Radiological analysis

Pelvic obliquity (POb) and Cobb’s angle of lumbar scoliosis (LS) in coronal plane were measured on the anterior-posterior XP images at pre-op and final follow up. In the coronal plane, 20mm of C7 plumb line shift is regarded as significant coronal imbalance [6]. Average spine length (C7 to pelvis) was 400mm in adult Japanese females, and 3.5 degrees of change in coronal plane corresponded to 20mm of change of C7 plumb line, therefore over 3.5 degrees of change in POb was regarded as ΔPOb(+). Over 10 degrees of maximum Cobb’s angle was regarded as LS(+) according to the SRS classification of adult scoliosis [6]. The change of lumbar scoliosis were classified into 3 subtypes; ΔLS(+), over 5 degrees of progress in LS, ΔLS(-), over 5 degrees of improvement in LS, and ΔLS(n), changes in LS within 5 degrees.

## Results

### 1. Measurement of radiological parameters

**Figure 1:**
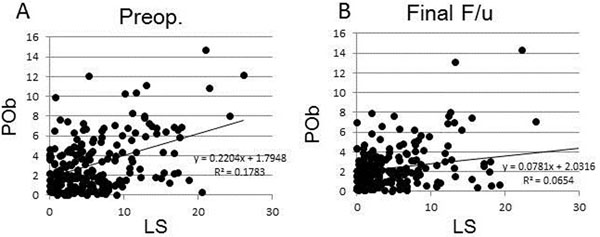
**Relationship between pelvic obliquity (POb) and lumbar scoliosis (LS).** A: preoperative results of POb and LS. There was low correlation between POb and LS (correlation coefficient = 0.34). B: Results at final follow up. There was no correlation between POb and LS (correlation coefficient = 0.25).

**Figure 2:**
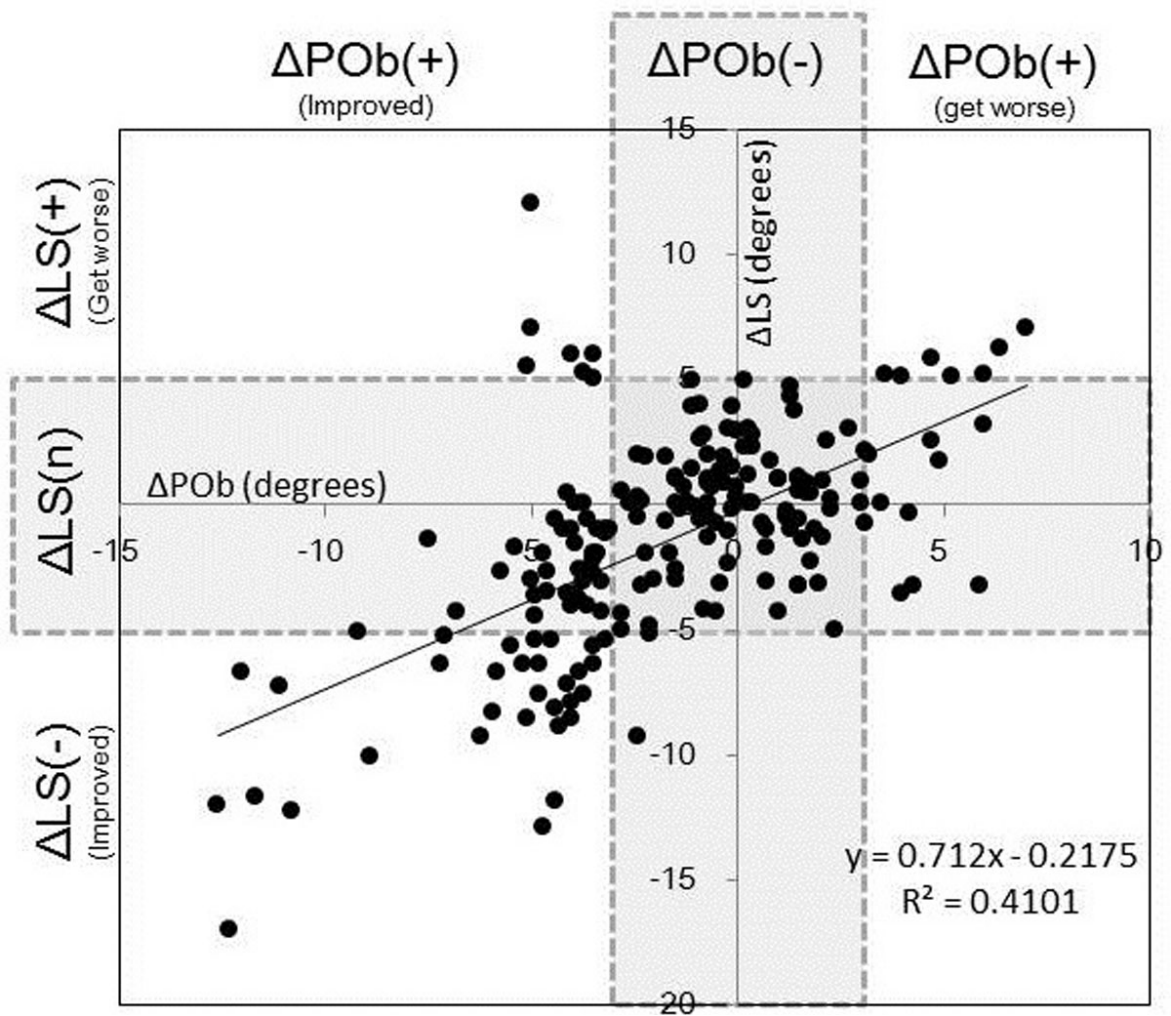
**Relationship between the changes of pelvic obliquity (ΔPOb) and lumbar scoliosis (ΔLS).** Positive correlation was observed betweenΔPOb andΔLS (correlation coefficient = 0.59). Horizontal axis means degree of the change of POb. Over 3.5 degrees was defined as significant (ΔPOb(+)). Vertical axis means degree of the change of LS. Over 5 degrees was defined as significant. ΔLS(-) means scoliosis was improved, ΔLS(n) means scoliosis did not changed, andΔLS(+) means scoliosis got worse.

The results of averaged POb showed that POb did not change from preoperative value (2.85±2.6) to postoperative value (2.40±2.0). LS did not change from preoperative value (6.03±6.2) to postoperative value (5.21±6.9). Degrees of preoperative POb had no correlation with preoperative LS, and POb at final follow up also had no correlation with LS (Fig. 1). On the other hand, change in degrees of POb was significantly correlated with the change in LS (Fig. 2). The 3^rd^ quadrant of the graph represented the cases showed the significant improvement of scoliosis (LS(-)) with decrease of pelvic obliquity. The cases in the 1^st^ quadrant showed the progression of scoliosis (LS(+)) with increase of pelvic obliquity.

### Classification of the change of coronal alignment of spine

**Figure 3:**
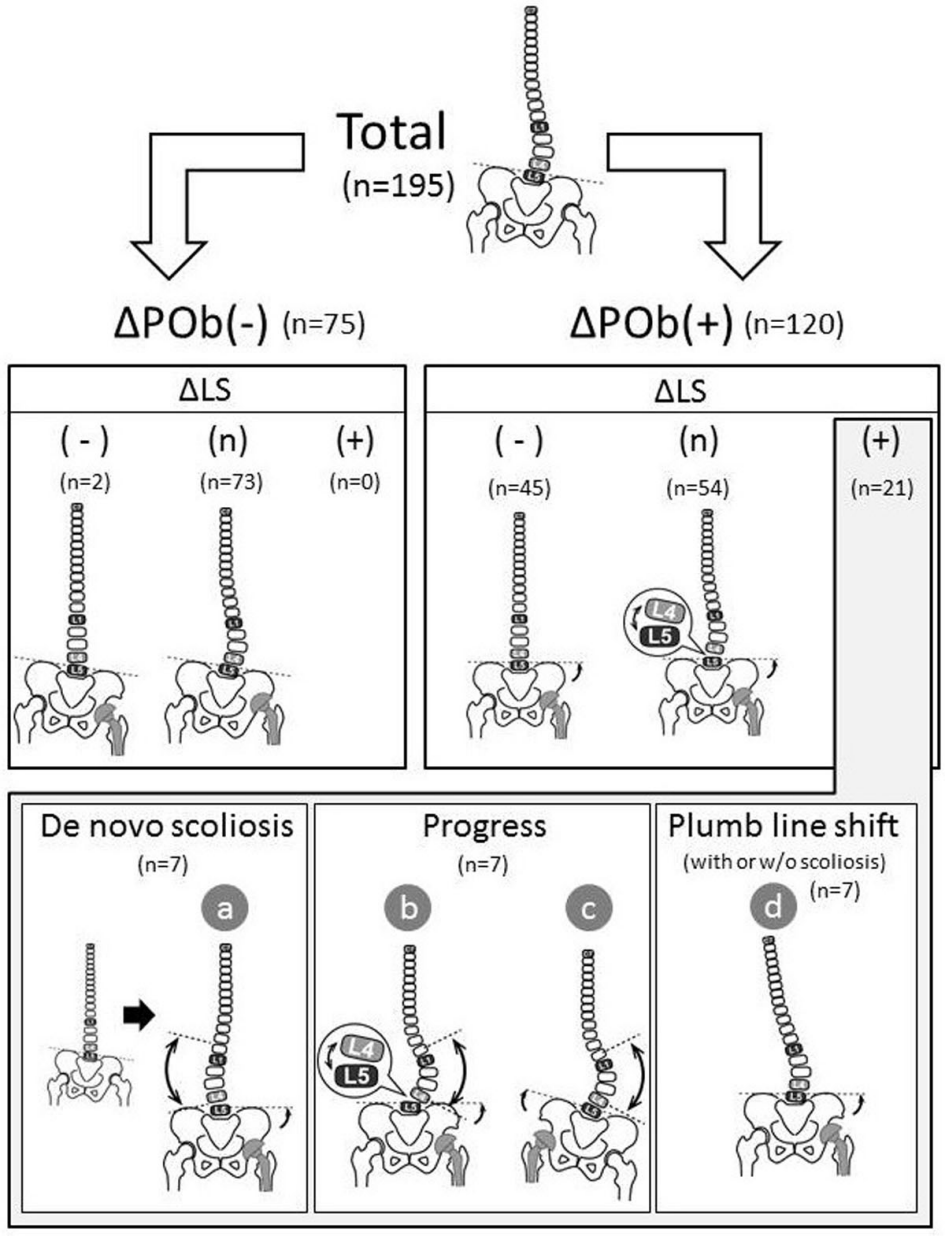
**Classification of the scoliosis change according to the POb change.** Classification of the scoliosis change according to the POb change. Seventy five of 195 did not exhibit POb change (ΔPOb(-)) and scoliosis did not progress. In 120 ΔPOb(+) patients, 45 showed improve of scoliosis (ΔLS(-)) and 54 showed compensation at lumbosacral junction without changing scoliosis (ΔLS(n)). Twenty one cases developed spinal imbalance or progressed scoliosis (ΔPOb(+),ΔLS(+)). A: 7 cases developed de novo scoliosis due to the rigid lumbosacral junction. B: 2 cases showed the over compensation at lumbosacral junction and developed lumbar scoliosis. C: 5 cases developed scoliosis due to the leg lengthening THA on the contralateral side. D: 7 cases developed plumb line shift due to the rigid lumbar scoliosis or lumbosacral junction.

Among all 195 patients, 75 patients did not exhibit POb change (classified as ΔPOb(-)) and 120 patients showed significant change in POb (ΔPOb(+)) (Fig. 3). No progression of lumbar scoliosis was observed in the 75 ΔPOb(-) patients. Among the ΔPOb(+) patients, 45 showed improvement of scoliosis (ΔLS(-)). A total of 54 patients compensated for the POb change by creating L4-S segmental wedging without developing de novo progression of lumbar scoliosis above L4 (ΔLS(n)). In the ΔPOb(+) patients, 21 patients showed progress or development of de novo scoliosis (ΔLS(+)). Seven of these 21 ΔLS(+) patients failed to compensate for the POb change at lumbosacral area and developed de novo lumbar scoliosis. Another 7 of these 21 ΔLS(+) patients who had rigid spine developed coronal trunk shift over 20mm. Five patients showed the progression of the scoliosis because of the POb increase by contralateral leg lengthening THA.

## Discussion

Coronal compensation of lumbar alignment after THA in the short term was classified in the present study. In the leg lengthening THA cases, 79% patients who had preoperative scoliosis showed the improvement of lumbar scoliosis according to the horizontalization of the pelvis. Among all 195 patients, 9.7% developed subclinical spinal imbalance and 1% developed severe low back pain due to the progression of lumbar scoliosis.

Previous studies reported that existence of pelvic obliquity did not always correlate with lumbar scoliosis [2-5]. Because compensation for imbalance of the pelvis could be done by not only the lumbar spine (L1-5), but also the lumbosacral junction or whole spine including thoracic spine, conventional X-ray evaluation using “lumbar scoliosis (L1-5 angle)” is not suitable for evaluating coronal balance in patients have tilted pelvis. Furthermore, Lee et al. [7] classified fixed pelvic obliquity after poliomyelitis into 8 patterns, and elucidated that lumbar scoliosis could develop in each side regardless of the direction of pelvic obliquity. The results of our study showed that the direction of lumbar scoliosis was not always coincident with the direction of pelvic obliquity, and the magnitude of lumbar curve did not correlate with degree of pelvic obliquity. These results suggested that lumbar scoliosis with tilted pelvis could be layered by idiopathic curve or be compensated by lumbo-sacral curve.

As regards the relationship between change in pelvic obliquity and change in lumbar scoliosis, there was a reproducible trend that decrease of pelvic obliquity improved lumbar scoliosis. Flexible scoliosis tended to be well improved by decrease of pelvic obliquity. 12 patients showed significant improvement of scoliosis 2 times greater than POb improvement. Even ridged scoliosis successfully compensated the change of pelvic obliquity by creating lumbosacral wedging at L4-S. However, over compensation at lumbosacral junction could be a potential risk for progression of L4 tilt and resultant coronal imbalance. Li et al. [8] reported that lateral tilt of lower instrumented vertebra over 8 degrees was a risk factor for developing postoperative coronal imbalance in Lenke type 5C scoliosis patients. Careful X-ray observation of coronal balance should be needed in case with postoperative increase in L4 tilt after leg lengthening THA.

## Conclusions

In conclusion, coronal balance of the spine was well compensated in 89.2% patient who received THA. Leg lengthening THA was considered to be safe as regards to the spinal alignment in coronal plane in short term. Few patients developed painful scoliosis and spinal imbalance, therefore preoperative evaluation of spinal flexibility and follow up using whole spine X-ray should be recommended. Further investigation is needed for elucidate the difference between preoperative exist degenerative scoliosis and leg lengthening THA induced scoliosis.

This is the extended abstract of IRSSD 2014 program book [9].

## Competing interests

The authors declare that they have no competing interests.

## Authors' contributions

YA have made contributions to conception and design, and have been involved in drafting the manuscript. KY and SA have made contributions to acquisition and analysis of data. SS and TM have made contribution to study design, revising the manuscript, and have given final approval of the version to be published.

## Declarations

Publication charges for this collection were funded by IRSSD 2014 Sapporo.

This article has been published as part of *Scoliosis* Volume 10 Supplement 2, 2015: Research into Spinal Deformities 9: Short Papers from the IRSSD 2014 Meeting. The full contents of the supplement are available online at http://www.scoliosisjournal.com/supplements/10/S2.
